# Factors Controlling Calanoid Copepod Biomass and Distribution in the Upper San Francisco Estuary and Implications for Managing the Imperiled Delta Smelt (*Hypomesus transpacificus*)

**DOI:** 10.1007/s00267-020-01267-8

**Published:** 2020-03-16

**Authors:** Scott Hamilton, Steve Bartell, James Pierson, Dennis Murphy

**Affiliations:** 1 Center for California Water Resources Policy and Management, 1017 L Street, Sacramento, CA 95814-3805 USA; 2 Highwood, Inc, 7610 Morganton Road, Greenback, TN 37742 USA; 3 University of Maryland, 2020 Horns Point Road, Cambridge, MD 21613 USA; 4 Graduate Program in Ecology, Evolution and Conservation Biology, University of Nevada, Reno, NV USA

**Keywords:** Sacramento-San Joaquin Delta, Calanoid copepod biomass, Environmental factors, Regression analysis, Delta smelt

## Abstract

Delta smelt struggle to persist in a dramatically altered estuarine environment. Complex and incompletely understood relationships between food availability, environmental stressors, other components of the species’ habitat, and the abundance of delta smelt impede the effective management and recovery of the species. The empirical modeling presented in this study quantitatively describes spatial-temporal biomass values of calanoid copepods, a key prey item for delta smelt, in relation to multiple potential controlling factors. The results underscore the role that river flows through the estuary have in determining prey availability, and demonstrate contributions of water temperature, salinity, and macronutrients in determining copepod biomass. The analysis also shows the importance of non-native, invasive bivalves in determining copepod biomass. Importantly, the analysis describes spatial-temporal shifts in the relative importance of modeled covariates across sampling locations in the Delta. Model results indicate that increasing flows in the fall of wetter years adversely affected copepod biomass, while increases in flows in the spring of drier years provided regional increases in biomass. The results of this analysis can inform resource management decisions and contribute to a comprehensive model that can meaningfully guide efforts to recover the imperiled delta smelt.

## Introduction

Conservation planners and resource managers are confronted with daunting challenges in their efforts to recover imperiled species in estuarine environments, which have been fairly described as the most anthropogenically degraded ecosystems on earth (Edgar et al. 2000). The dimensions of the ecological disturbances affecting estuaries, ranging from upstream reservoirs that modify rates and patterns of freshwater inflow, to contaminants and introduced species that disrupt food webs and displace native species, combine to frustrate ecosystem restoration efforts. The upper San Francisco Estuary, including the Sacramento-San Joaquin Delta, on California’s coastal midsection is experiencing those and other sources of ecosystem disturbance and ecological stress, presenting the quintessential multi-dimensional conservation challenge, one where multiple endangered and threatened species struggle to survive on a landscape that has endured a century and a half of development and re-engineering.

Effects of environmental alterations on the resident fish communities in the upper San Francisco Estuary have been dramatic. Two native fish species are now extinct, nine fish species and salmon runs are listed by federal and state governments as endangered or threatened, and all remaining native species in the upper estuary persist at reduced and declining numbers (Moyle et al. 2010). The imperiled delta smelt, narrowly distributed and endemic to the system, is the focus of numerous and controversial management efforts. The delta smelt and its estuarine habitat appear compromised in every discernable ecological dimension; much of its habitat has been altered or destroyed and water quality conditions are frequently unsuitable in its remaining habitat. Invasive fishes prey on delta smelt and compete with it for food resources. The preferred zooplankton prey of delta smelt are decreasingly available in portions of the smelt’s range and through phases of its annual life cycle.

Modifications of river flows using releases from upstream reservoirs provide an opportunity to enhance the performance of desired ecological attributes in the estuary. The specific mechanisms by which river flows can contribute to meeting conservation objectives are often poorly articulated in conceptual ecological models. This leads to erratic and unpredicted results from directed management actions. The imperfect understanding of the ecological system and linkages among delta smelt and its essential resources has led resource planners to conservation management by proxy (Murphy and Weiland 2019). Asserting that the surface extent of the low-salinity zone in the estuary is a “surrogate indicator” of delta smelt habitat has led to a management directive—provide more freshwater outflow through the highly regulated system to contribute to the recovery of the delta smelt. That simple prescription has failed thus far to benefit the delta smelt, whose numbers continue to decline. It has come at substantial economic and social cost as water available for agricultural and municipal uses has been greatly curtailed. Moreover, while the conservation efforts for delta smelt has focused on the ecological importance of through-Delta flows (Kimmerer 2002b; Lund et al. 2015), emerging analyses have failed to find a direct relationship between abundance of delta smelt and freshwater input into the system (Kimmerer et al. 2009, 2013). While multiple physical and biotic factors have been identified as potentially having deleterious effects on delta smelt in conceptual ecological models, the estimated importance of those factors in determining delta smelt numbers has varied widely in multivariate analyses (MacNally et al. 2010; Thomson et al. 2010; Maunder and Deriso 2011; Miller et al. 2012). Those investigations contributed to the conundrum facing resource managers—how can a recovery strategy be designed and implemented when causes for delta smelt decline cannot be substantiated?

One possible answer can be drawn from studies that consider the delta smelt’s zooplankton prey, which include several species of calanoid copepods. Reduced biomass of those copepods in some years and in certain seasons has been identified as important in determining the distribution and abundance of delta smelt (Bennett 2005; Kimmerer et al. 2008; IEP 2008). More recently, food shortages in certain years and seasons have been identified as the factor limiting the abundance of delta smelt (Hamilton and Murphy 2018). Copepod availability across the geographic range of delta smelt has declined demonstrably, with food shortages being exacerbated in recent decades by invasions of non-native invertebrates and fish (Carlton et al. 1990; Mahardja et al. 2016; Kimmerer and Thompson 2014). The best available science strongly indicates that a recovery strategy for delta smelt should focus on improving food availability, and that requires an understanding of the environmental factors that influence the distribution and abundance of calanoid copepods.

The purpose of the present study was to identify and quantify factors affecting the biomass and distribution of calanoid copepods in the Sacramento-San Joaquin Delta. An empirical model was developed to evaluate candidate management actions that could contribute to conservation and recovery of delta smelt. This study answers several questions that link the availability of delta smelt with freshwater flows through the Delta: what environmental factors cause copepod biomass to change throughout the year? How do river flows influence the distribution and biomass of calanoid copepods? And, can river flows be modified to improve the availability of the copepods to delta smelt, and thereby contribute to smelt recovery? This investigation considers how modification of river flows might affect calanoid copepod biomass and addresses two flow-related management actions: (1) increasing in the Sacramento River outflow in the autumn of wetter years; and (2) increasing in outflow in spring in the Sacramento and San Joaquin rivers in drier years.

## Methods

In order to inform the development of a spatially and temporally explicit empirical model, we begin by reviewing the ecological circumstances of calanoid copepods in the upper San Francisco estuary. We then identify and assemble data, depict graphically the distribution of the copepods, and parameterize two versions of an empirical model that can address different management questions. Each model version is quantified for 7 months at 12 locations. We utilize those specifications to build a flow-based simulation model to evaluate the above-mentioned candidate management actions.

### The Ecological System

**Fig. 1:**
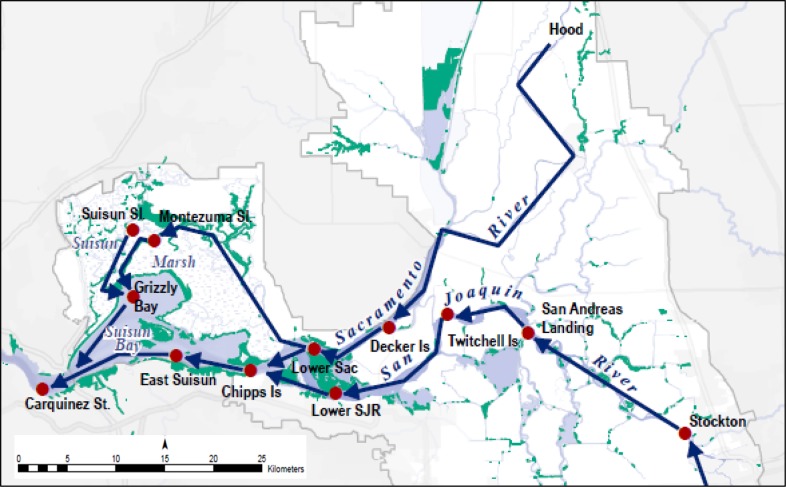
The study area in the upper San Francisco Estuary with locations of monitoring stations. Red dots show locations of stations from which data are used in this study. Blue lines indicate the normal direction of river flows. Green shading shows the locations of tidal and emergent wetlands. Vernalis, which is not shown on the map, lies ~40 km south of Stockton on the San Joaquin River. The area to the east of Chipps Island is referred to as the Sacramento-San Joaquin Delta (the Delta), and the area to the west of Chipps Island as Suisun Bay and Marsh

Habitat for delta smelt is limited to the Sacramento-San Joaquin Delta and Suisun Bay and Suisun Marsh, and in wetter years, the Napa River and Carquinez Strait. This area is the spatial domain for our study (Fig. 1). The Napa River can provide habitat in certain years, and the north Delta likely has a year-round delta smelt population, but neither of those regions have monitoring stations that record data that are necessary for this study and were not included in our analysis.

Delta smelt primarily prey upon calanoid copepods, although adult delta smelt will utilize additional prey when available (Sommer and Mejia 2013). *Eurytemora affinis* early in spring and *Pseudodioptamus forbesi* in summer and fall have been identified as copepod species most frequent in the diets of rearing delta smelt (IEP MAST 2015). The copepods are also prey for other small fishes, jellyfish, shrimp, and predatory copepods. These crustaceans have a life cycle of ~2 weeks. A wide diversity of factors has been implicated in regulating copepod species composition and numbers. This includes phytoplankton availability, competition for resources and predation on all life stages, toxic blue-green algae occurrences, floodplain inundation, water temperature, and salinity (Cryer and Townsend 1988; Orsi and Mecum 1996; Mauchline 1998; Sobczak et al. 2002; Mueller-Solger et al. 2006; Grosholz and Gallo 2005; Ger et al. 2009; Brucet et al. 2010; Greene et al. 2011; Kimmel 2011; Bollens et al. 2014; Durand 2015; O’Rear and Moyle 2018).

Phytoplankton, particularly diatoms, are important food sources for copepods. Greater production and growth of phytoplankton occur in shallow and shoal areas that are not light limited (Cloern et al. 1983). Local diatom biomass appears to be greatest under moderate through-Delta flow regimes when these algae are neither discharged downstream by higher flows nor sinking at rates that exceed vertical mixing with low-velocity flows. The spatial and temporal distribution of phytoplankton exhibits variability at three scales, reflecting recurrent seasonal environmental influences, interannual variability in environmental conditions (including variability in river flows), and trends or regime shifts resulting from system perturbations (Jassby et al. 1993; Cloern and Jassby 2010). Declines in chlorophyll *a*, an indicator of phytoplankton biomass, have been associated with the establishment of invasive the Asian clam (*Corbula amurensis)*, residence time, and estuarine outflow (Hammock et al. 2019). Kimmerer et al. (1998) noted that observed maximum abundances of *E. affinis* and *P. forbesi* were associated with specific salinity ranges, suggesting copepod ability to maintain position in the estuary despite net outflow.

Both tidal and diurnal movements of copepods have been well documented (Kimmerer and McKinnin 1987; Mauchline 1998). However, Kimmerer et al. (1998) did not find that copepods were more abundant in the water column during the flood tide than ebb tides, contrary to findings in other estuaries (Kimmerer and McKinnin 1987). Kimmerer et al. (2018a, b) indicated that the copepod community in the Delta and adjacent portions of the estuary experiences spatial subsidies and losses, and concluded the principal mechanism by which flow affects the *P. forbesi* population is the apparent transport of copepods downstream. River flows then are of primary interest in this study, influencing among other things, residence times in sub-basins, not only for copepods, but also for the lower-trophic organisms on which they depend.

### Data Sources and Availability

A number of environmental factors may influence copepod biomass. Relevant data for those and other factors were obtained from two primary monitoring efforts in the estuary—the Interagency Ecological Program (IEP) zooplankton survey and the environmental monitoring program (EMP).

Calanoid copepod abundance at multiple life stages has been monitored in the Delta in the IEP zooplankton survey since 1972, along with chlorophyll *a* concentrations, although the sampling schema have changed over time. Since 1994, the zooplankton survey has sampled 19 stations monthly, including 17 fixed stations and 2 stations that vary in location in relation to salinity (where electrical conductivity at the bottom of the channel is 2 and 6 mS/cm).

The EMP gathers data on physical and chemical factors in water samples and operates 17 stations within the study area. There were 14 locations where both zooplankton and EMP sampling were conducted (Supplementary Table S-1). Of the 17 locations initially considered, only 12 locations on the Sacramento and San Joaquin rivers had sufficient zooplankton data for analysis.

The availability of data for environmental covariates varied widely (see Supplementary Table S-3). Due to absence of zooplankton data, stations at Hood and Vernalis were not included in the study. Mysids were not considered a predator of calanoid copepods. The data for shrimp, other than mysids, jellyfish and protozoa were erratic in quality, appeared unreliable, and were not included in the study. The use of chlorophyll *a* may not accurately represent phytoplankton biomass. The relationship between phytoplankton biomass and chlorophyll *a* can vary (Cloern et al. 1995; Jakobsen and Markager 2016), depending on local conditions. However, Mueller-Solger et al. (2002) found differences in growth rates of juvenile *Daphnia magna* (a cladoceran) in delta habitats could be attributed to differing chlorophyll *a* concentrations. This suggests that the use of chlorophyll *a* may serve as a useful proxy for the phytoplankton. Data on abundance of small fish likely to prey on copepods were drawn from trawl surveys conducted throughout the year (CDFW—https://www.wildlife.ca.gov/Regions/3). Data were collected on age-0 striped bass, longfin smelt, delta smelt, pacific herring, northern anchovy, threadfin shad, and Mississippi silversides. The catch per unit effort (CPUE) for these fish were summed to provide an index of predator pressure. The invasive silversides were also considered separately because of their dramatic increase in abundance over the last 20 years (Mahardja et al. 2016). Flow data were obtained from Dayflow (DWR—https://water.ca.gov/Programs/Environmental-Services/Compliance-Monitoring-And-Assessment/Dayflow-Data) using proximate locations, and Fairfield monthly precipitation data were used as a proxy for flows in Suisun Slough. Nutrient data were obtained from the EMP and all other data were obtained from the zooplankton survey (Supplementary Table S-2).

To develop an initial understanding of the distribution of calanoid copepod biomass throughout the estuary, and how that varies with hydrology, we summarized graphically the available survey data by month, location and hydrologic year type. The designation of hydrologic year types was obtained from California Department of Water Resources (http://cdec.water.ca.gov/cgi-progs/iodir/wsihist).

### Empirical Model

To understand the nature of the relationship between the environmental covariates and calanoid copepod biomass (hereafter, copepod biomass), we plotted data for each covariate against copepod biomass at each location in each month. For several factors, there were weak associations (*R*^2^ < 0.1) with copepod biomass in any month at any location. These factors included turbidity, dissolved silica, dissolved oxygen, and a predator-pressure index. Water column stratification consistently showed a weaker association with copepod biomass than salinity. Nitrogen concentration and river flows frequently demonstrated nonlinear relationships with copepod biomass. Phosphorus concentration was either weakly associated with copepod biomass, or where phosphorus demonstrated an association with copepod biomass, the association between nitrogen and copepod biomass generally was stronger. Given these results, turbidity, dissolved silica, dissolved oxygen, stratification, phosphorus and predator pressure were eliminated as covariates from the subsequently constructed empirical model.

To develop the empirical model, we specified an equation that related the influence of covariates to copepod biomass at each site, *s*, in each month, *t* Eq. (1).

1 $$\begin{array}{l}\mathrm{log}\left( {Z_{t,s}} \right) = \beta _0 + \beta _1\mathrm{log}\left( {Z_{t - 1,s}} \right) + \beta _2\mathrm{log}\left( {Z_{t,u}} \right) + \beta _3B_y \\ + x\beta _4\mathrm{log}\left( {E_{t,s}} \right) + \beta _5\mathrm{log}\left( {E_{t,s}} \right)^2 + \beta _6T_{t,s} + \beta _7C_{t,s} + \beta _8S_{t,s} \\ + \beta _9A_{t,s} + \beta _{10}N_{t,s} + \beta _{11}N_{t,s}^2 + \beta _{12}F_{t,s} + \beta _{13}X_y\end{array}$$

where *Z* is copepod biomass (μgC/m^3^), *B* is a dummy variable equal to 1 after the introduction of the Asian clam in 1986, *E* is estuarine river flows (previous average 30-day flow at proximate Dayflow location in cubic feet per second), *T* is water temperature (°C), *C* is chlorophyll *a* (μg/L), *A* is ammonia (mg/L as *N*), *N* is dissolved nitrate (mg/L as *N*), *F* is CPUE of predatory silversides (#/10,000 m^3^), *S* is surface salinity (µS/cm) and *X* is a trend variable with an annual time step *y*. Data sources are provided in Supplementary Table S-2. The subscript u denotes upstream stations, with some locations having more than one upstream location flowing into it. Note we use the terms “upstream” (east) and “downstream” (west) relative to the station of interest.

Two versions of the model were constructed. Both versions were derived by using the minimization of adjusted *R*^2^ for the model selection criterion. Version 1, potentially useful for addressing specific flow-related management actions, included only the first six covariates in Eq. (1) (previous and upstream copepod biomass, non-native bivalves that compete with copepods for food, river flows, and temperature).

Version 2 includes all the covariates in Eq. (1). This version would be useful for evaluating management actions that focus on factors other than river flow. Estimated coefficients for some covariates sometimes had signs inconsistent with previous work (see Table S-2). Additionally, it did not seem plausible that bivalves could increase biomass of calanoid copepods, nor that upstream biomass could detract from downstream biomass. In instances where these estimated coefficients had signs that were not supported by our understanding of these relationships, the corresponding covariates were removed from the equation and coefficients of the remaining covariates were re-estimated. Variables that produced such “incorrect” signs were identified with an “X” in the results tables. The coefficients in all models were estimated using the statistical package XLStat (Addinsoft 2019).

Each version of the empirical model produces 84 fitted equations (one for each of seven months for 12 locations; each month-location combination is referred to as a sub-model). Each sub-model was estimated separately. To test for autocorrelation in sub-models, we checked if the Durbin–Watson statistic was significantly different from two when there was no lagged dependent term in a sub-model, and the significance of the *h* statistic (*h* > 1.645) when the sub-model included a lagged dependent term (Rao and Miller 1971). When serial autocorrelation was detected we re-estimated the equations using Cochrane-Orcutt estimation (Cochrane and Orcutt 1949; XLStat, Addinsoft 2019).

### Management Application

To consider the possible influence of flow-related management actions on copepod biomass, Version 1 of the empirical model was transformed into a simulation model. Equation (1) includes as factors upstream copepod biomass and prior month copepod biomass. That is, actions that influence upstream or prior copepod biomass can influence subsequent or downstream copepod biomass. To incorporate this temporal and spatial connectivity into the simulation model, actual covariate data on copepod biomass is substituted with estimates from prior equations. Two hypothetical management actions were considered: (1) an increase of 4700 cfs in the Sacramento River flow in September and October in wetter than average years (subsequently referred to as a fall-flow action), and (2) an increase in flow of 1000 cfs in April and May in the Sacramento and San Joaquin rivers in drier than average years (subsequently referred to as a spring-flow action). The first management action is similar to one component of the “reasonable and prudent alternative” expected to benefit delta smelt. It was proposed as a directed management action in a biological opinion targeting the fish (USFWS 2008). The second action was stimulated by a need to increase delta smelt recruitment in Suisun Marsh—understood to be a region that should contribute substantially to delta smelt numbers. The period from 1972 to 2015 was simulated using historical water temperature data. Historical river flow data were increased according to the two described management actions. The bivalve covariate for all years was set equal to one, reflecting the current circumstance of invasive clam establishment in the estuary.

## Results

**Fig. 2:**
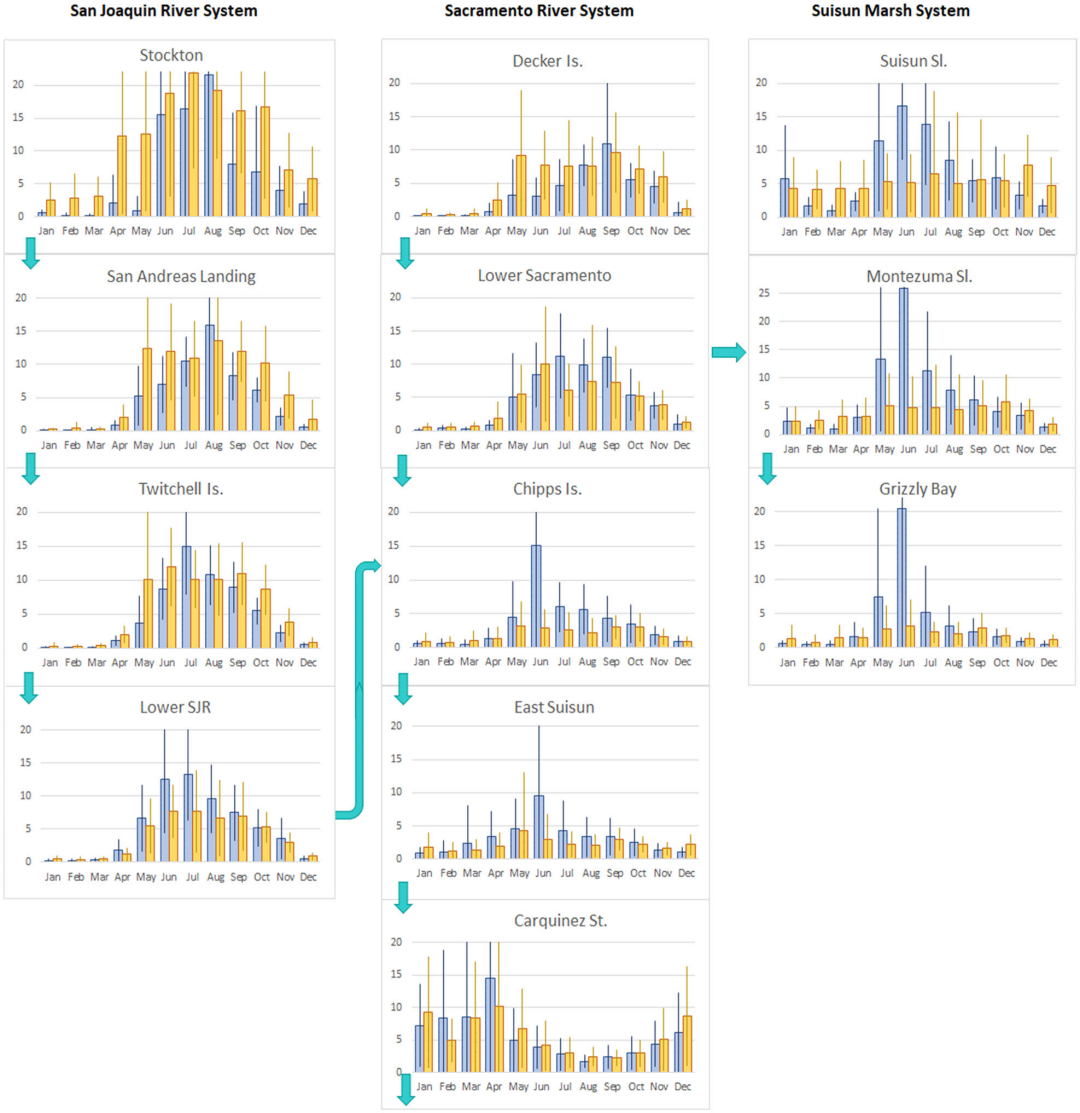
Average biomass of calanoid copepods by month and location in each river system. Arrows indicate influence of river flows from one location to the next. Units for vertical axes are mgC/m^3^. Blue columns are copepod biomass averages of water-year types classified as “wet” and “above normal.” Orange columns are copepod biomass averages of water-year types classified as below “normal,” “dry,” and “critical.” Vertical bars indicate one standard deviation from the mean

Plotting calanoid copepod biomass by month, location, and hydrologic year type shows spatial and temporal patterns in the biomass of copepods (Fig. 2). Copepod biomass tended to decline from upstream to downstream locations with higher values downstream in wetter years. The differences in biomass between wet and dry years diminish as the year progresses. In the Suisun Marsh system, biomass in wet years was dramatically greater in May through July with little differences during the rest of the year. When comparing river systems, the San Joaquin River had greater copepod biomass in both wet and dry years. The seasonal trend in Carquinez Strait, which only provides habitat for delta smelt in wet years, is the reverse of that for the rest of the system, with higher copepod biomass in the spring diminishing into the summer. There thus are regional differences in seasonal copepod biomass independent of hydrology. However, differences in hydrology appear to have a significant influence on productivity in the spring and summer at certain sites, and on the extent to which copepod biomass moves downstream.

**Fig. 3:**
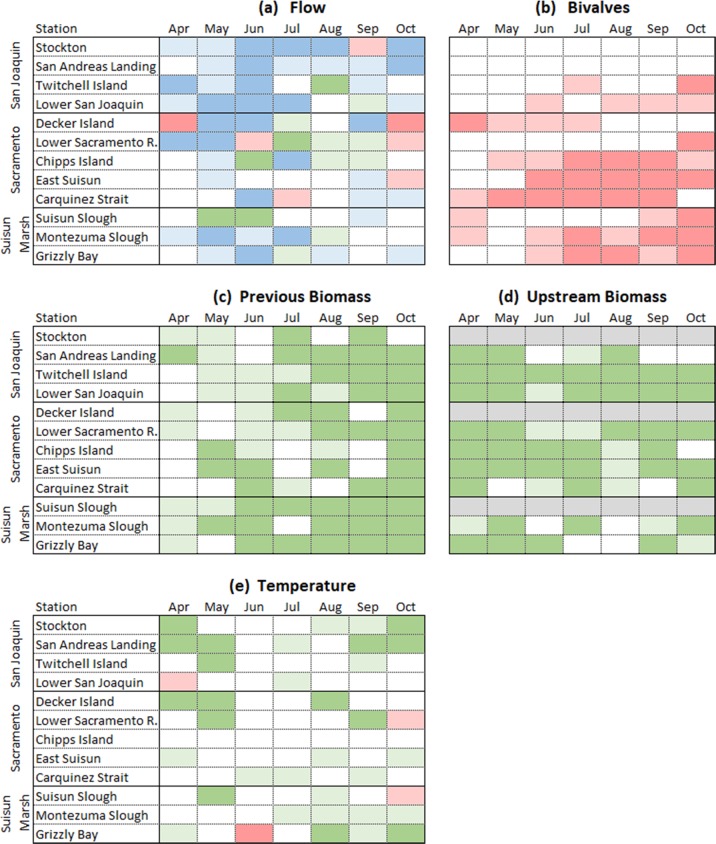
Depiction of the influence of five environmental factors—(**a**) flow, (**b**) bivalves, (**c**) previous biomass, (**d**) upstream biomass, and (**e**) temperature—on copepod biomass across survey locations and seasons. Green-colored boxes indicate that the factor has a positive association with copepod biomass, rose-colored boxes indicate a negative association, and blue boxes a nonlinear association. Light gray boxes indicate no data were available. Boxes that have darker shading represent statistically significant relationships in Version 1 sub- models. Boxes with lighter shading represent relationships that were not significant. Boxes with no shading represent factors that were not included in Version 1 sub-models

The influence of hydrology observed in Fig. 2 was also apparent in the regression analysis for Version 1 of the model (Fig. 3a and Supplementary Table S-4). The large number of statistically significant flow coefficients (darker blue squares in Fig. 3a) suggest that river flows are an important factor influencing the distribution and biomass of copepods. The contribution of river flows tended to be very high in the spring and summer of the more easterly locations. The influence of river flows was generally less in the autumn and in the westerly locations of East Suisun Bay and Carquinez Strait. The relationship of river flow to copepod biomass at Decker Island and Stockton frequently was not monotonic. Copepod biomass increased over a range of river flows, and then decreased as higher flows transported copepod biomass downstream.

The influence of clams was significant and strongest in the latter half of the year in regions in Suisun Bay (Fig. 3b). The influence of temperature was frequently significant in the Delta in the spring and autumn, and in the shallow water of Grizzly Bay in the autumn.

We considered the importance of in situ copepod production versus upstream subsidy (Fig. 3c, d). Both are apparent in the data. In situ production was significant at multiple times and places especially from July onwards in the Delta and from June onwards in Suisun Bay and Suisun Marsh. Upstream production was also widely significant for most months in the Delta and through July in regions in Suisun Bay and Suisun Marsh.

The goodness of fit (*R*^2^) averaged over months was lowest in Suisun Slough (average *R*^2^ = 0.54) where flow data were unavailable and exceeded 0.7 on the San Joaquin River near Stockton and Twitchell Island, and in Carquinez Strait (Supplementary Table S-4). The fitted equations provided greater explanatory power for the San Joaquin system than the Sacramento system.

Version 2 of the empirical model still showed river flow, in situ copepod production, and upstream copepod subsidies to be contributing to copepod biomass, as were bivalves at the westerly sites. Other environmental factors were only important at certain locations and times (Supplementary Fig. S-1). Significant associations of copepod biomass with chlorophyll *a* mostly occurred during April through June in the Sacramento River and Suisun Marsh. Electrical conductivity, a proxy measure of salinity, frequently had a negative association with biomass in the northern Delta and Suisun Bay, except in October. Ammonia had a significant negative association in June and July on the Sacramento system and in May at Twitchell Island and East Suisun. The correlation of nitrogen with copepod biomass, when it occurred, was generally positive or nonlinear. The potential influence of predatory Mississippi silversides was rarely evident but did occur in the central part of the system (Lower Sacramento, Lower San Joaquin, and Chipps Island) in August and September. After accounting for other factors, trends in copepod biomass were generally positive in May and in the autumn, but negative in April. Grizzly Bay showed positive trends in summer and autumn, but only after accounting for the introduction of the Asian clam.

**Fig. 4:**
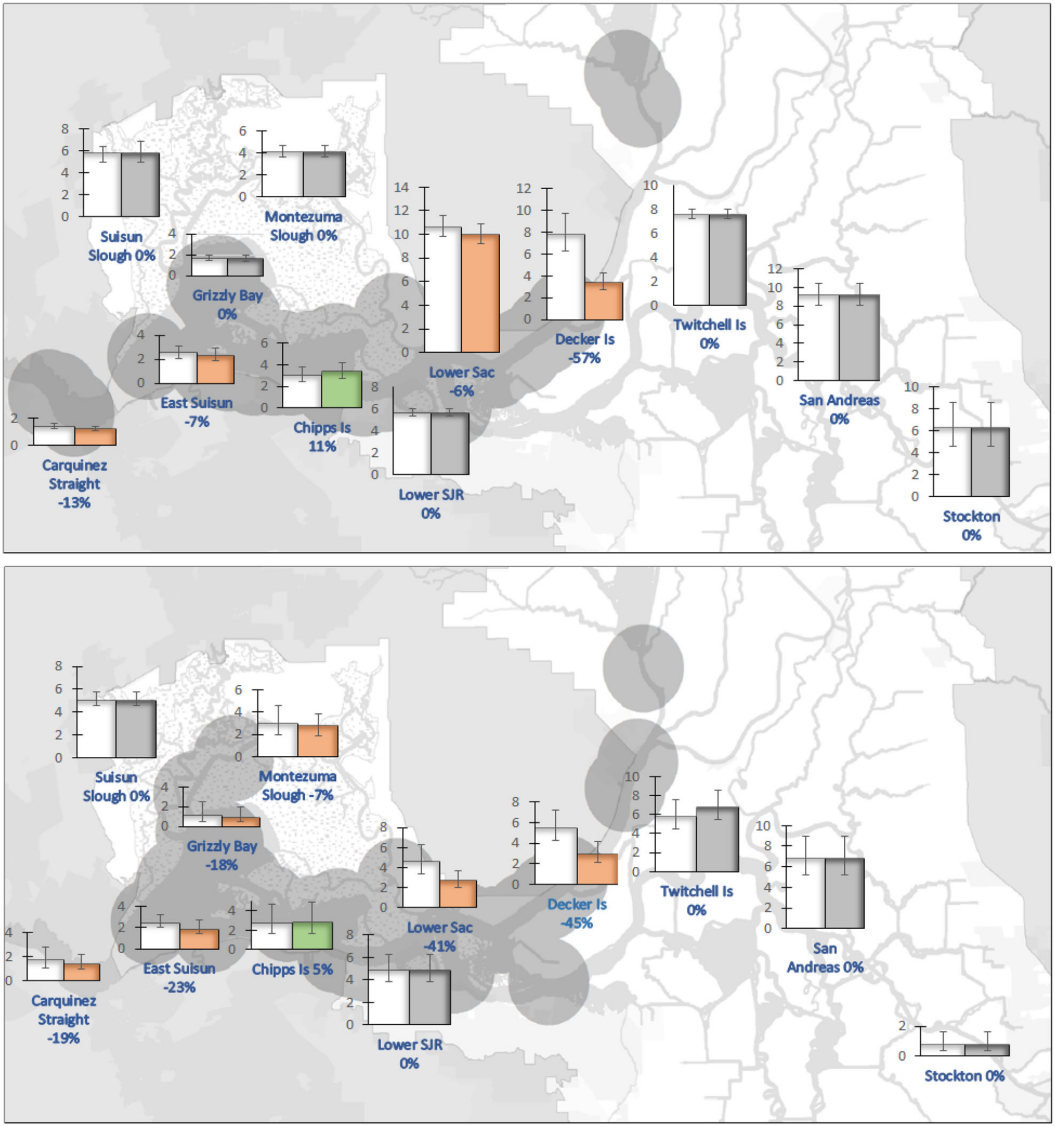
Projected calanoid copepod biomass (mgC/m^3^) of a 4500 cfs increase in Sacramento river flows in September (top) and October (bottom) of wetter years. Orange columns reflect decreases in copepod biomass, green columns reflect increases, and gray columns show no change in copepod biomass compared with the no-action circumstance (white columns). Vertical bars indicate the mean square error of the estimate. Biomass of calanoid copepods in the San Joaquin River did not show change because the fall-outflow management action when implemented was confined to the Sacramento River. Gray circles indicate stations where on average 90% of delta smelt were sampled (normalized so each year is weighted equally) in wetter than average years in the Fall Midwater Trawl since 1987

The simulated fall-flow action predicted decreases in copepod biomass at every location in the Sacramento River except Chipps Island, where copepod biomass increased from 4 to 7% (Fig. 4). The greatest decreases in copepod biomass were predicted to occur upstream in October (24% at Decker Island and 36% in the Lower Sacramento River), which may manifest as food deficits for delta smelt that occupy those areas in the fall.

**Fig. 5:**
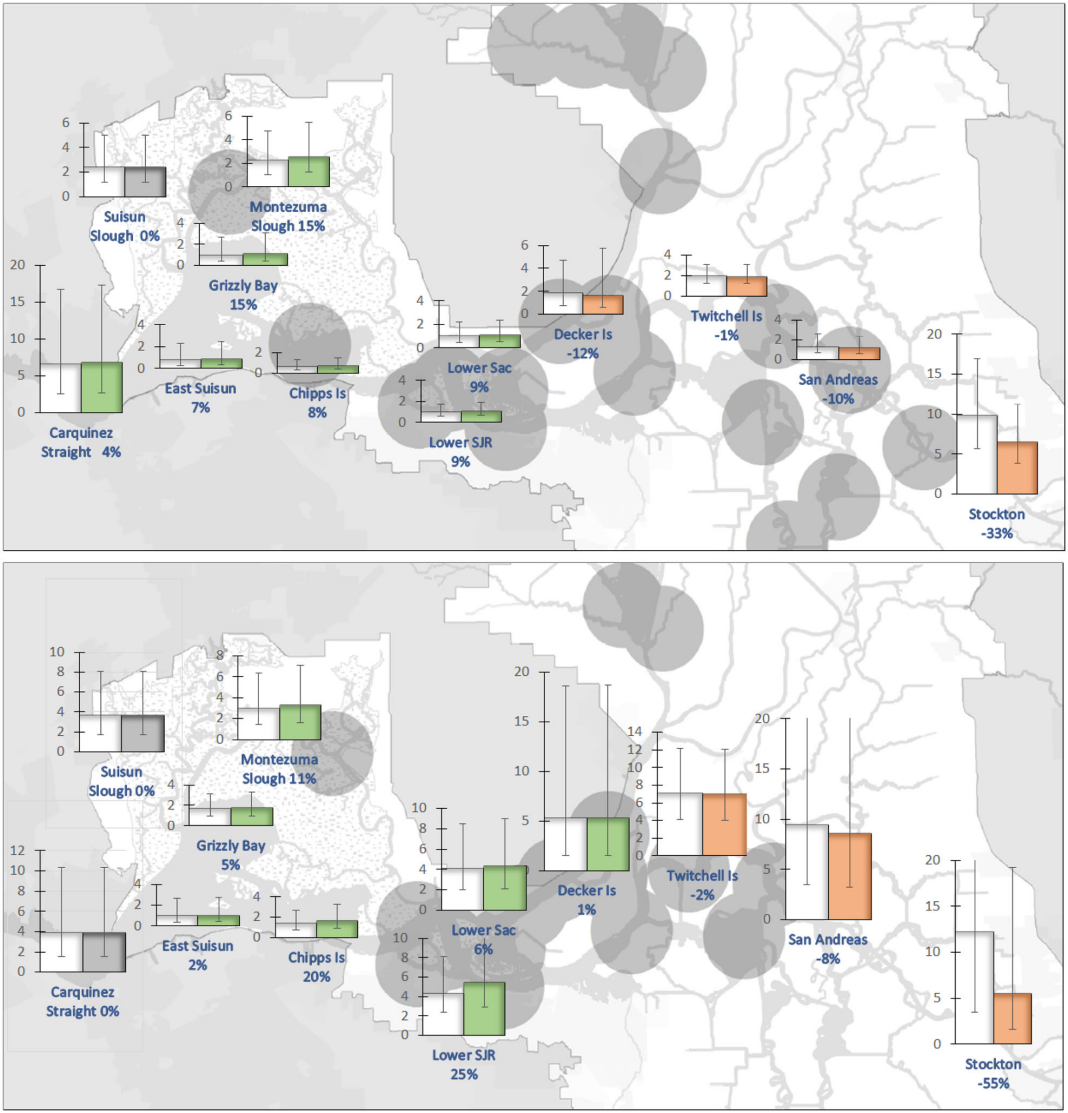
Projected calanoid copepod biomass (mgC/m^3^) of a 1000 cfs increase in Sacramento and San Joaquin river flows in April (top) and May (bottom) of drier years. Orange columns reflect decreases in copepod biomass, green columns reflect increases, and gray columns show no change in copepod biomass compared with the no-action circumstance (white columns). Vertical bars indicate the mean square error of the estimate. Gray circles indicate stations where on average 90% of delta smelt were sampled (normalized so each year is weighted equally) in drier than average years in the 20 mm survey since 1995

A simulated spring-flow action predicted a decrease in the copepod biomass at upstream locations (Decker Island and Stockton). Unlike the fall-flow action, the spring-flow action was predicted to provide dispersed downstream benefits—copepod biomass increases of 11 to 29% in April and 5 to 23% in May at locations between the lower Delta locations and Montezuma Slough (Fig. 5). The benefits of such an action to delta smelt remain to be investigated; the action was predicted to both increase and decrease copepod biomass at locations where delta smelt are found in April. In May, the action was predicted to increase biomass in areas where delta smelt are usually observed.

**Fig. 6:**
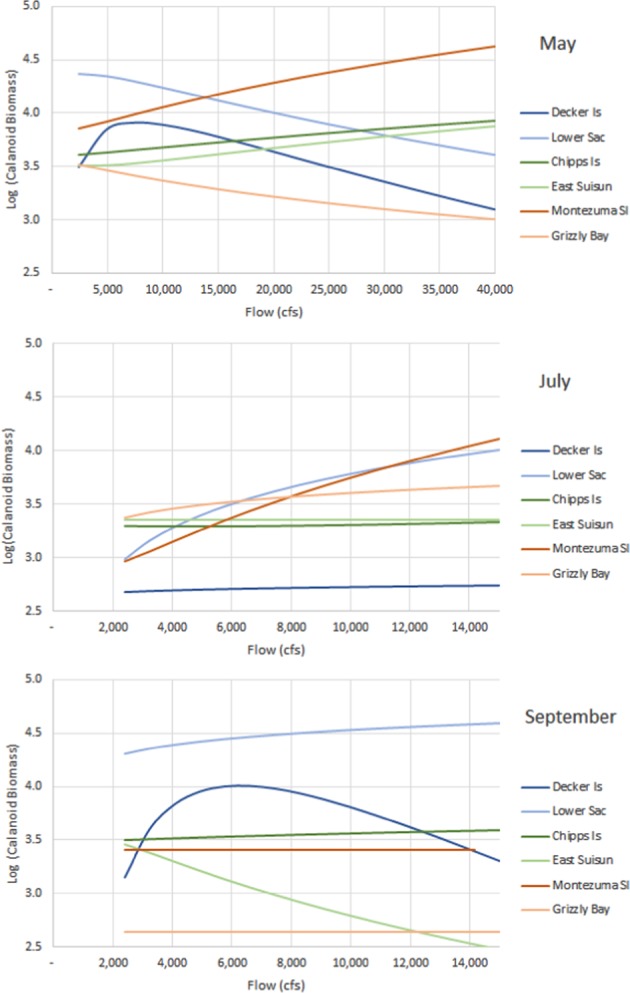
Estimated response of calanoid biomass to flows in the Sacramento River system depicting variation by location and month

To better understand the influence of the potentially nonlinear interactions between river flow and copepod biomass, we plotted the modeled relationship derived from the parameterization of Eq. (1) for three example months: May, July, and September (Fig. 6). The nonlinear relationship was most pronounced at Decker Island. Increased river flows in May quickly dispersed biomass, decreasing it at Decker Island and the Lower Sacramento River, and increasing it at Chipps Island, East Suisun Bay, and Montezuma Slough. In July river flows are weaker and copepod dispersion not so pronounced. Increasing flows in July decrease biomass at Decker Island and increases it at Lower Sacramento River and Montezuma Slough. Biomass for Chipps Island and East Suisun Bay appears largely unresponsive to changes in flows in July. By September, the copepod responses at Decker Island and Lower Sacramento River are repeated, but biomass at Montezuma Slough at that time appears unresponsive to changes in flows.

## Discussion

Failed efforts to reverse the decline in numbers of the endemic and imperiled delta smelt in the upper San Francisco Estuary have led conservation planners to question assumptions about the environmental factors that limit the size of the fish’s population, including the availability of calanoid copepods, its primary prey. The results of this present study contribute to the understanding of how environmental factors influence the productivity and distribution of calanoid copepods in the estuary.

### Calanoid Copepod Production

The empirical analysis and simulation modeling presented here demonstrate the importance of river flows and other environmental factors in determining copepod biomass in spatial context. While river flows provide a major contribution to total organic carbon into and through the Delta (Jassby et al. 1993, 2003), its influence on copepods is less well understood (Kimmerer 2002a, b). In the Chesapeake Bay estuary, the biomass of a *Eurytemora* species is positively correlated with river flow (North and Houde 2006; Martino and Houde 2010), a relationship that has been attributed to higher phytoplankton availability, resulting from increased nutrient loads imported into the system with greater flows (Kimmel et al. 2009). Anomalously high flow into Chesapeake Bay has been shown to have spatially distinct impacts on different zooplankton taxa (Roman et al. 2005).

Our results make evident that the relationship between copepod biomass and river flows in the upper San Francisco Estuary is complex—more outflow is not necessarily better. The extent of the downstream transport of copepod biomass appears to shift with the strength of the flows and the season. Copepod biomass in the Sacramento River system has a statistically significant response to changes in flow in spring and summer in many regions but has a statistically less significant response in the autumn. Changes in river flows may be effective in redistributing copepods early in the year, but they appear to be less effective as the year progresses.

Other factors that influence copepod biomass include: bivalves that negatively impact calanoid biomass in the second half of the year in Suisun Bay and Suisun Marsh, nitrogen in some months, negative impacts of ammonia in the Sacramento River system in May through July, and negative impacts of higher salinity in most regions and months, except in October when higher copepod biomass is associated with higher than average levels of salinity. Salinity, as well as concentrations of nitrogen and ammonia, are influenced by river flows (Domagalski et al. 2000; IEP MAST 2015). Relationships between N and P concentrations and phytoplankton growth are realistically described by nonlinear Monod functions (Lehman et al. 1975; Bothwell 1988; Son and Fujino 2003). Similar to river flows into the estuary, the relationship between copepod biomass and nitrogen availability is frequently nonlinear, suggesting an as-yet-unidentified optimal nitrogen level for estuarine waters that is correlated with river flows (understanding that greater flows apparently dilute nitrogen levels).

Water temperature has a positive relationship with copepod development, rates of growth, size, and reproduction (Pierson et al. 2016; Hirst and Forster 2013; Hirst and Kiørboe 2014; Bunker and Hirst 2004; Lloyd et al. 2013; Miller et al. 1977). The availability of data at finer spatial-temporal resolution, such as that on water temperature, might permit incorporation of known nonlinear and interactive relationships between certain covariates and copepod productivity (Schneider 1992; Peters and Downing 1984).

### Management Implications

The results of our analysis and modeling provide several insights into the potential management of copepod production to increase food availability for delta smelt. First, changes in river flows, achieved by modifying releases from upstream reservoirs, can redistribute copepod biomass. Frequently, biomass increases in one area of the estuary, while decreasing elsewhere. But the general trend is that copepod biomass is lesser at downstream locations than upstream locations (Fig. 2). Second, the effect of changes in river flow on the overall biomass and redistribution of copepods varies by month and location (Fig. 6). The effectiveness of managing flows for the purpose of distributing copepods appears to decline as the calendar year progresses (Fig. 6). Increased river flows later in the year tend to decrease biomass throughout the estuary by moving copepods from upstream locations into Suisun Bay where the effects of clam grazing are pronounced (Fig. 3). Third, reservoir regulation designed to benefit delta smelt must consider where the fish are likely to be located in a particular season, noting that those locations vary with hydrologic year type. Increased San Joaquin River flows in May in years of lower flow, for example, can usefully transport copepod biomass towards areas occupied by delta smelt (Fig. 5). However, in autumns of wetter years, increased river flows may move copepods away from areas of greater delta smelt density and reduce copepod abundance throughout the estuary (Fig. 4).

In a dynamic physical-chemical and biological system, such as the upper San Francisco Estuary, copepod productivity is complex and multiple factors operate simultaneously to influence it (IEP MAST 2015). Previous investigations have promoted management of the spatial and temporal extent of the low-salinity zone, a putative “surrogate indicator” of delta smelt habitat (Feyrer et al. 2007; USFWS 2008; Nobriga et al. 2008; Feyrer et al. 2011; Castillo 2019). Those studies contend that delta smelt benefit from the expanded extent of the low-salinity zone when it is located downstream in the upper estuary. Other investigations suggest that the quality, rather than the extent, of habitat for delta smelt is more important. Evaluation of habitat quality is complex and requires consideration of factors other than outflow through the estuary, including the sensitivity of delta smelt to a range of abiotic conditions (Manly et al. 2015), the availability of the phytoplankton prey of calanoid copepods, and spatial and temporal distribution of other zooplankton and phytoplankton (Jassby et al. 1993; Jassby et al. 2002; Kimmerer et al. 2002a; Cloern and Jassby 2008; Cloern and Jassby 2012; Kimmerer and Thompson 2014; Hamilton and Murphy 2018; Kimmerer et al. 2018a, 2018b). The results from this study suggest that directed management actions that push the low-salinity zone downstream, thereby expanding the extent of the low-salinity zone, will not be helpful for delta smelt if it is associated with a decline in the biomass of copepods (and see Murphy and Weiland 2019).

Management actions unrelated to flow may impact copepod biomass. A primary source of ammonia in the upper San Francisco Estuary is effluent from the Sacramento Waste Water Treatment plant (SWRCB 2008; Parker et al. 2010). Modifications to the treatment plant that could reduce ammonia levels are already planned (Regional San 2016). Another source of nitrogen is runoff from agricultural fields adjoining streams flowing into the Delta. The Central Valley Regional Water Quality Control Board has programs to reduce runoff and nitrogen loading (https://www.waterboards.ca.gov/centralvalley/water_issues/irrigated_lands/outreach_brochure.pdf). The results presented here suggest that the success of those programs in reducing nitrogen inputs could indirectly reduce copepod biomass and affect prey availability for delta smelt (e.g., Köhler et al. 2005; Gutierrez et al. 2016).

### Path Forward to Effective Management

Copepod productivity is a primary factor limiting the recovery of delta smelt (IEP MAST 2015; Hamilton and Murphy 2018). The location of delta smelt varies by life stage and through-Delta flows that vary by year and season (e.g., Murphy and Hamilton 2013; Hobbs et al. 2019). Thus, the focus of management actions could simply be to improve the circumstances where the fish are most commonly found; that is, to improve the spatial and temporal overlap of the distribution of delta smelt with suitable levels of copepod biomass. Given apparent historic mismatches, managers should consider that the fish are frequent in and adjacent to Suisun Marsh and the north Delta during spawning and larval life stages in the late winter and spring, and in the northern arc, from North Suisun east to the lower Sacramento River, in the summer and fall (recognizing some variance around those locations due to hydrology—also see Merz et al. 2011). The use of flow regulation to benefit delta smelt by increasing or decreasing flows to maintain copepod biomass above critical thresholds in areas occupied by delta smelt requires thoughtful implementation.

Based on the results presented here, a path forward for effective management of delta smelt and its copepod prey might include several essential elements—(a) quantifying the minimum prey thresholds for delta smelt by month, recognizing that the bioenergetic requirements of smelt change with bodyweight and water temperature, (b) adjusting monitoring efforts targeting delta smelt and zooplankton to estimate relative differences in copepod biomass between regions to assess whether copepod biomass overlaps with the distribution of delta smelt, (c) determining whether reservoir releases can be used to redistribute copepods to increase prey availability in target areas to meet minimum threshold levels required to support delta smelt, and (d) implementing the actions in an adaptive management framework to assess and improve the effectiveness of managed flows.

The importance of our empirical analysis of the complex, spatial-temporal relationships between copepod biomass and environmental covariates lies in developing appropriately scaled management actions that benefit delta smelt by focusing on key environmental factors at the right places and right times.

## Supplementary information

Supplementary Information
