# Supplementary material for: Factors Controlling Calanoid Copepod Biomass and Distribution in the Upper San Francisco Estuary and Implications for Managing the Imperiled Delta Smelt (Hypomesus transpacificus)
Source: Environ Manage. 2020 Mar 16;65(5):587–601. doi: 10.1007/s00267-020-01267-8 (PMC7145783; doi:10.1007/s00267-020-01267-8)
Supplement: Supplementary file 1 — Supplementary Information [file 267_2020_1267_MOESM1_ESM.docx]

**SUPPLEMENTAL MATERIAL**

*Additional Information Pertaining to Data Sources and Availability*

As discussed in the body of the paper, a number of environmental factors may influence copepod biomass. Relevant data were obtained from two primary monitoring efforts in the estuary -- the Interagency Ecological Program (IEP) zooplankton survey and the Environmental Monitoring Program (EMP). Fish catch and abiotic data are collected in a number of trawl surveys. Correspondence between stations, compared to station-sites in Figure 1, is presented in Table S-1. Source of data are presented in Table S-2. Data availability is summarized in Table S-3.

Calanoid copepod biomass at multiple life stages has been monitored in the Delta in the IEP zooplankton survey since 1972, along with Chlorophyll *a* concentrations. Three gear types are used to sample different size classes of zooplankton. They include a pump for sampling microzooplankton <1.0 mm long, including rotifers, copepod nauplii, and adult copepods of the genus *Limnoithona*; a modified Clarke-Bumpus (CB) net for sampling mesozooplankton 0.5-3.0 mm long, including cladocerans, copepodids, and adult copepods; and a macrozooplankton net for sampling zooplankton 1-20 mm long, including mysid shrimp. Samples are fixed in formalin and transported to the California Department of Fish and Game Laboratory in Stockton for processing. (<https://www.wildlife.ca.gov/Conservation/Delta/Zooplankton-Study>)

The designation of hydrologic year types was obtained from California Department of Water Resources (<http://cdec.water.ca.gov/cgi-progs/iodir/wsihist>).

**Table S-1** Locations in the study area and proximate zooplankton, EMP, and fish survey stations. See Figure 1 for a map of the site locations

| **Site Location** | **Zooplankton Station** | **EMP Station** | **20MM Stations** | **FMWT Stations** | **Upstream Zoop Stations** |
| --- | --- | --- | --- | --- | --- |
| Vernalis | None | C10A | None | None | None |
| Stockton | NZ092 | P8 | 910,912 | 910, 911, 912 | None |
| San Andreas Landing | NZ086 | D26 | 815,906 | 815, 904,  905, 906 | SJR Near Stockton |
| Twitchell Is | NZD16, NZ080, NZ082 | D16 | 812 | 809, 810,  811, 812 | San Andreas Landing |
| Lower SJR | NZ074 | D12 | 804 | 802, 803,  804, 805 | Twitchell Is |
| Hood | None | C3A | None | 735, 736 | None |
| Decker Is | NZ064 | D22 | 705,706,707 | 705, 706, 707 | None |
| Lower Sacramento River | NZ060 | D4 | 513 | 512, 513, 514 | Decker Is |
| Chipps Is | NZ054 | D10 | 508 | 506, 507,  508, 509 | Lower Sac Decker Is |
| East Suisun Bay | NZ048 | D8 | 504 | 502, 503, 504 | Chipps Is |
| Carquinez Strait | NZD06, NZ020, NZ004, NZ042 | D6 | 405,411,418 | 405, 407, 409, 410, 414 | Grizzly Bay  East Suisun Bay |
| Suisun Slough | NZS42 | NZS42 | None | None | None |
| Montezuma Slough | NZ032 | NZ032 | 606 | 605, 606 | Lower Sac |
| Grizzly Bay | NZ028 | D7 | 602 | 601, 602,  603, 604 | Suisun Sl Montezuma Sl |

FMWT – Fall Midwater Trawl Survey

EMP – Environmental Monitoring Program

20MM – 20mm Survey

**Table S-2** Sources of data


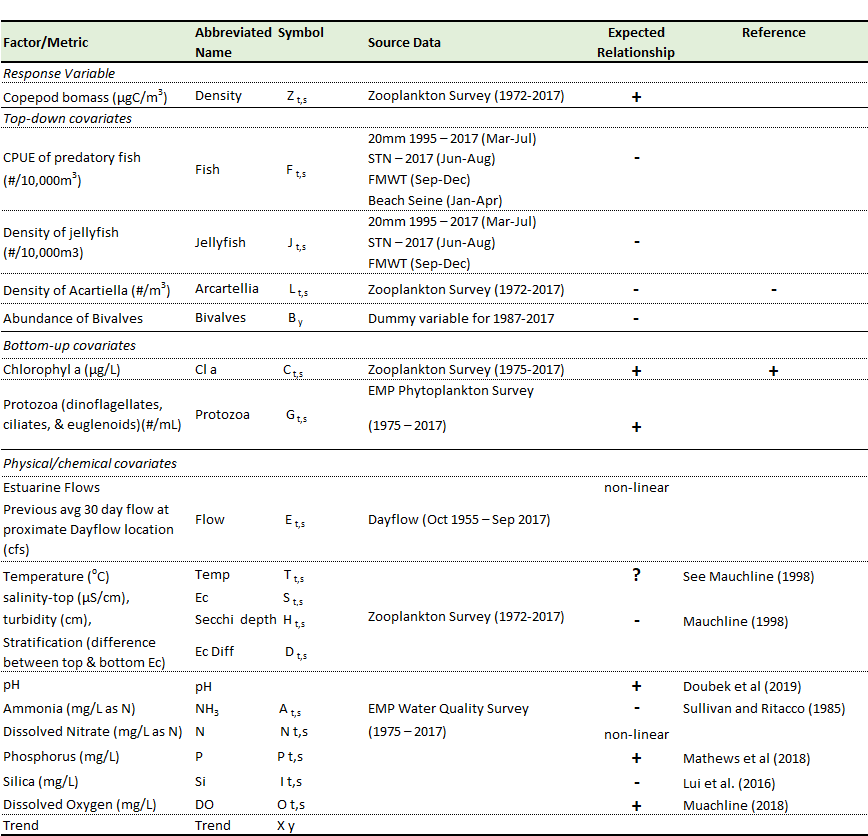


CPUE – catch per unit effort

STN – Summer Tow-Net Survey

FMWT – Fall Mid-water Trawl Survey

EMP – Environmental Monitoring Program

20mm – 20mm Survey

Subscript designations: (y) year, (t) month, (s) station.
Fairfield precipitation data obtained from <https://www.ncdc.noaa.gov/cdo-web/review>

**Table S-3** Data availability for factors influencing the distribution and biomass of calanoid copepods in the upper San Francisco estuary. Rose-colored cells indicate factors and locations with insufficient data. Tan cells indicate factors and locations with data limitations, and green cells indicate factors and locations with data that are generally adequate. Number ranges indicate years of availability.

| **Location** | **EMP station** | **Zoo-plankton** | **Fish** | **Shrimp & Jellyfish** | **Chlorophyll *a*** | **Protozoa** | **Flows** | **Abiotic Conditions** | **Nutrients** |
| --- | --- | --- | --- | --- | --- | --- | --- | --- | --- |
| Vernalis | C10A | None |  |  |  |  |  |  |  |
| Stockton | P8 | 72-17 | Apr-Jun 95-17 Jul, Sep-Dec 72-17 Aug spotty | 2007+ | 72-17 | 75-17 | Vernalis | 72-17 | 79-15 |
| San Andreas Landing | D26 | 72-17 | Apr-Jun 95-17 Jul, Sep-Dec 72-17 Aug spotty | 2007+ | 72-17 | 75-17 | QWest | 72-17 | 79-15 |
| Twitchell Is | D16 | 72-17 | Apr-Jun 95-17 Jul, Sep-Dec 72-17 Aug spotty | 2007+ | 72-17 | virtually none | Qwest | 72-17 | 79-95 |
| Lower SJR | D12 | 72-17 | Apr-Jun 95-17 Jul, Sep-Dec 72-17 Aug spotty | 2007+ | 72-17 | spotty | Qwest | 72-17 | 79-95 |
| Hood | C3A | None |  |  |  |  |  |  |  |
| Decker Is | D22 | 72-17 | Apr-Jun 95-17 Jul, Sep-Dec 72-17 Aug spotty | 2007+ | 72-17 | virtually none | Rio Vista | 72-17 | 79-95 |
| Lower Sac | D4 | 72-17 | Apr-Jun 95-17 Jul, Sep-Dec 72-17 Aug spotty | 2007+ | 72-17 | 75-17 | Rio Vista | 72-17 | 79-15 |
| Chipps Is | D10 | 72-17 | Apr-Jun 95-17 Jul, Sep-Dec 72-17 Aug spotty | 2007+ | 72-17 | virtually none | Outflow | 72-17 | 79-95 |
| East Suisun Bay | D8 | 72-17 | Apr-Jun 95-17 Jul, Sep-Dec 72-17 Aug spotty | 2007+ | 72-17 | 75-17 | Outflow | 72-17 | 79-15 |
| Carquinez Strait | D6 | 72-17 | Apr-Jun 95-17 Jul, Sep-Dec 72-17 Aug spotty | 2007+ | 72-17 | spotty | Outflow | 72-17 | 79-15 |
| Suisun Slough | NZS42 | 72-17 | none |  | ?? | virtually none | Outflow | ?? | none |
| Montezuma Slough | NZ032 | 72-17 | Apr-Jun 95-17 Jul, Sep-Dec 72-17 Aug spotty | 2007+ | 72-17 | virtually none | Outflow | 72-17 | none |
| Grizzly Bay | D7 | 72-17 | Apr-Jun 95-17 Jul, Sep-Dec 72-17 Aug spotty | 2007+ | 72-17 | 75-17 | Outflow | 72-17 | 79-15 |

**Table S-4** Regression results showing estimated coefficients of covariates and significance at each location and month. Numbers in bold indicate coefficients with P values less than 0.05. A dash indicates the covariate was not included in the preferred model; “X” indicates the covariate had an incorrect sign and was excluded from the regression.


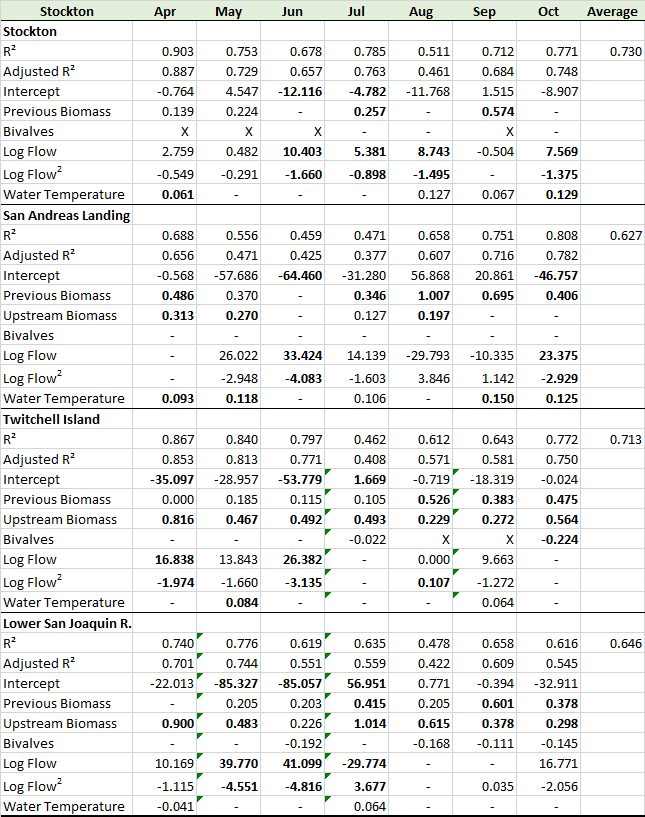


**Table S-4** Continued


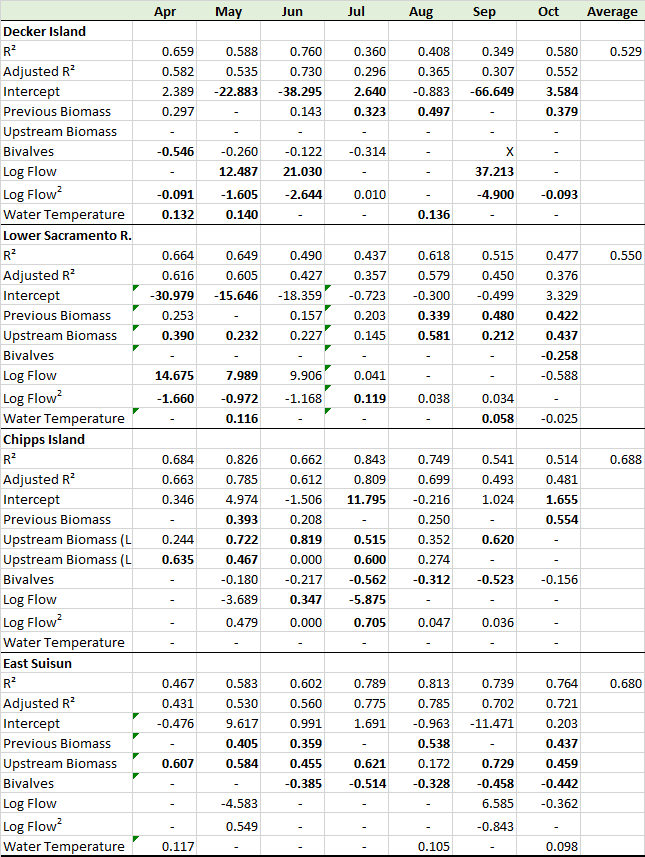


**Table S-4** Continued


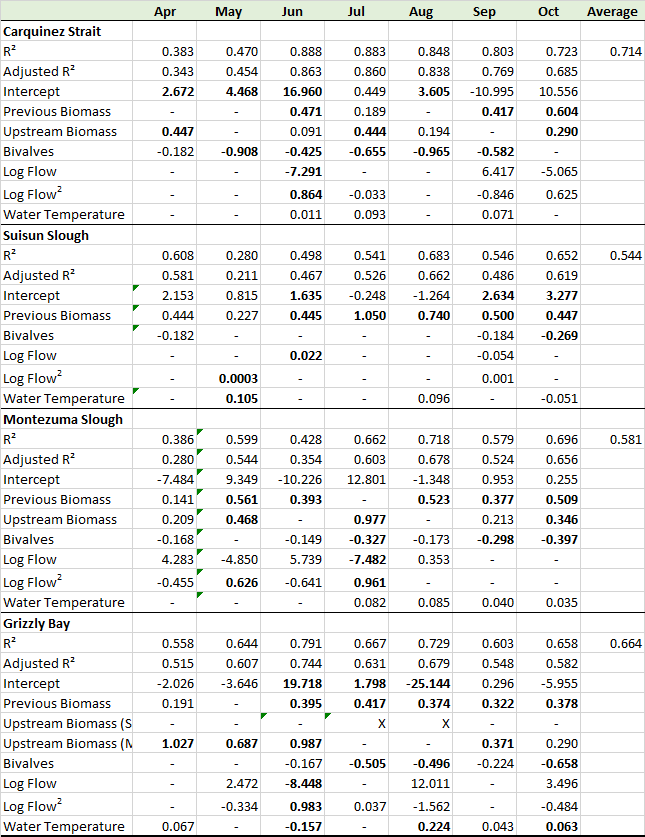


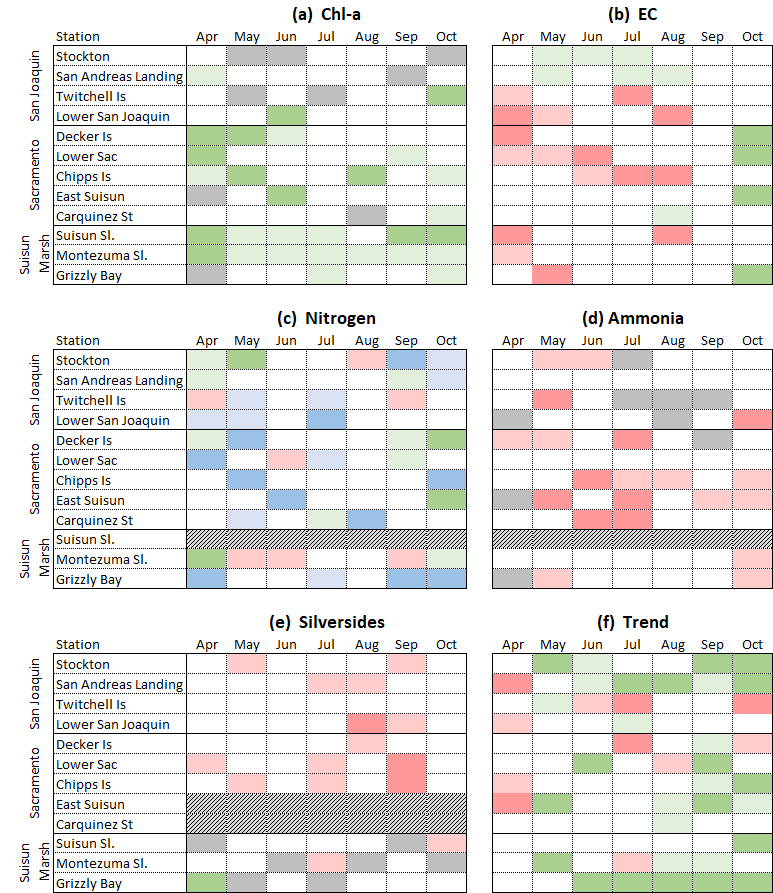


**Fig S-1.** Depiction of the covariates that were not included (unshaded), included (light shading) and significant (dark shading) in version 2 of sub-models. Green shading indicates a positive association with calanoid biomass, pink a negative association, blue a non-linear association, light grey that data were unavailable and dark grey that the covariate had an incorrect sign and was omitted from the sub-model.
